# Chaotic decorrelation of Globus Pallidus by periodic forcing: a possible mechanism for the therapeutic effects of deep brain stimulation

**DOI:** 10.1186/1471-2202-12-S1-F3

**Published:** 2011-07-18

**Authors:** Charles J Wilson, Bryce Beverlin, Theoden Netoff

**Affiliations:** 1 Department of Biology, University of Texas at San Antonio, San Antonio, TX 78249, USA; 2 School of Physics and Astronomy, University of Minnesota, Minneapolis, MN 55455, USA; 3 Department of Biomedical Engineering, University of Minnesota, Minneapolis, MN 55455, USA

## 

High frequency (~100 Hz) stimulation of the subthalamic nucleus is an effective therapy for the symptoms of Parkinson's disease. Presumably, stimulation partly normalizes a pathological change in rate or pattern of neuronal activity in the output neurons of the globus pallidus. The rate effects of subthalamic stimulation are paradoxical, because firing rate in the globus pallidus is increased in Parkinson's disease, and subthalamic input is excitatory. Parkinson's disease is also accompanied by changes in globus pallidus cell firing pattern. Pallidal neurons are autonomous single-spiking oscillators that fire continuously and usually show no periodic bursting. They fire independently of each other, having flat cross-correlations. In Parkinson's disease the cells burst at 5-10/s and are highly correlated [1]. Rubin and Terman [2] have proposed that subthalamic stimulation entrains globus pallidus cells interfering with the low frequency bursting. In their model, the synchrony of globus pallidus cells should be high, because all cells are entrained by the same stimulus. The periodic nature of deep brain stimulation is inconsequential in their model.

A recent clinical study has shown that the periodicity of deep brain stimulation is critical to its effectiveness [3]. Poisson stimulation at the same rate was much less beneficial. This observation, coupled with the frequency dependence of DBS effectiveness, suggests that disruption of low frequency bursting may not be the only mechanism of action. Globus pallidus cells are oscillators, so it is possible that periodic stimulation may not only suppress low frequency bursting, but also disrupt synchrony by driving the cells into chaotic firing patterns.

We examined the range of stimulus frequencies and amplitudes required to chaotically desynchronize pallidal neurons. Oscillating pallidal cells were represented by their phase resetting curves. We used a type 1 phase resetting curve similar to that measured from model pallidal neurons [4]. The variance of the phase resetting curve was estimated as in Ermentrout et al [5] and used to form a stochastic phase map. The stationary distribution of latencies and the phase resetting curve were used to calculate Lyapunov exponents for both hyperpolarizing and depolarizing stimulation, and compared to the synchronizing or desynchronizing effect of stimulation on a population of simulated pallidal neurons that were weakly synchronized by common noisy input. Powerful synchronization and desynchronization were evoked at different frequencies as predicted by the sign of the Lyapunov exponents. Chaotic decorrelation in response to depolarizing stimulation occurred at frequencies similar to those known to be most effective for deep brain stimulation clinically.

We suggest that in addition to its effects on low frequency oscillations in the pallido-thalamic projection, deep brain stimulation decorrelates the activity of pallidal neurons by driving them into a chaotic firing pattern. When the stimulus is randomized the chaotic response is destroyed, explaining why it is less effective. This chaotic effect may be essential to the clinical benefit of stimulation, being responsible for its frequency sensitivity and the requirement for periodicity in the stimulus pattern.
